# The association between neutrophil percentage-to-albumin ratio and cardiovascular disease: evidence from a cross-sectional study

**DOI:** 10.3389/fcvm.2025.1557507

**Published:** 2025-05-14

**Authors:** Xinfu Huang, Yuqing Zhang, Wanting Hao, Xue Wu, Peng Yang

**Affiliations:** ^1^ Second Clinical Medical College, Guizhou University of Traditional Chinese Medicine, Guiyang, China; ^2^ Department of Rheumatology and Immunology, The Second Affiliated Hospital of Guizhou University of Traditional Chinese Medicine, Guiyang, China

**Keywords:** neutrophil percentage-to-albumin ratio, cardiovascular disease, cross-sectional study, NHANES, predictive marker

## Abstract

**Background:**

Cardiovascular disease (CVD) is a leading cause of death and disability worldwide. Available studies suggest that inflammation and nutritional status play a key role in the development of CVD. As a new combined indicator of inflammation and nutritional status, the neutrophil percentage-to-albumin ratio (NPAR) may be important in CVD prediction.

**Objective:**

This study investigated the association between NPAR and CVDs such as heart failure, coronary heart disease, angina pectoris, and stroke. It aimed to confirm the validity of NPAR as a potential biomarker of CVD using data from the National Health and Nutrition Examination Survey (NHANES).

**Methods:**

This study used a cross-sectional study design that analyzed the neutrophil percentage, albumin levels, and CVD diagnostic information of 12,165 adults. Multifactorial logistic regression modeling was employed to explore the association between NPAR and CVDs such as heart failure, coronary heart disease, angina pectoris, and stroke, while the nonlinear relationships were examined via restricted cubic spline. In addition, subgroup analyses were performed to assess the effect of age, sex, and race on the association between NPAR and CVD.

**Results:**

Our findings suggested that higher NPAR levels were significantly associated with an increased odds of CVD events. Specifically, each NPAR unit increase was associated with a 3% higher odds of a CVD event (OR = 1.03, 95% CI: 1.01–1.06). Individuals in the highest NPAR quartile displayed a significantly higher odds of heart failure (OR = 1.66, 95% CI: 1.18–2.34, *p* = 0.0035)and stroke (OR = 1.74, 95% CI: 1.28–2.36, *p* = 0.0004) than those in the lowest quartile. Subgroup analyses showed a more pronounced association between NPAR and CVD in women (OR = 1.04, 95% CI: 1.00–1.08, *p* = 0.0499), hypertensive patients (OR = 1.04, 95% CI: 1.01–1.07, *p* = 0.0154), and diabetic patients (OR = 1.05, 95% CI: 1.01–1.09, *p* = 0.0178).

**Conclusion:**

The study demonstrated that as a comprehensive indicator of inflammation and nutritional status, NPAR could effectively predict CVD occurrence. Although the clinical application value of NPAR requires further validation, it shows promise as a novel biomarker for early CVD screening and prevention.

## 1 Introduction

Cardiovascular disease (CVD) is a leading cause of death and disability worldwide. According to the World Health Organization (WHO), about 17.5 million people die of CVD each year, accounting for 31% of all global deaths (1). Cardiovascular diseases, including coronary heart disease, angina pectoris, and stroke, primarily involve pathological mechanisms such as atherosclerosis, inflammatory response, and metabolic abnormalities, and heart failure is a clinical consequence of these diseases (2). CVD incidence has continued to rise in recent years due to lifestyle changes and population aging, posing a significant challenge to public health and the healthcare system (3).

Although a large number of studies have explored the CVD risk factors, such as hypertension, high cholesterol, diabetes, and smoking, existing research still displays significant limitations (4, 5). First, traditional risk factors are insufficient to clarify all the mechanisms behind CVD. For example, one study found that a considerable number of CVD cases remained unexplained even after controlling traditional risk factors (6). This highlights a critical gap in our understanding of the multifactorial nature of CVD and suggests that additional, non-traditional factors may play a significant role in its development and progression. Second, although the roles of inflammation and nutritional status in CVD development have received increasing attention, the related biomarker studies remain insufficient (7, 8). Inflammation is now recognized as a key driver of CVD pathogenesis, yet commonly studied inflammatory markers such as leukocytes and C-reactive protein (CRP) exhibit limitations regarding sensitivity and specificity (9). Similarly, nutritional status is intricately linked to CVD risk, but traditional markers often fail to capture the complex interplay between nutrition and inflammation (10). Moreover, there is a lack of integrated biomarkers that can comprehensively assess both inflammation and nutritional status simultaneously, which is crucial for a more holistic understanding of the odds of developing CVD. Therefore, developing new approaches to accurately predict and manage CVD is essential.

The neutrophil percentage-to-albumin ratio (NPAR) has attracted considerable attention in recent years as a new integrated indicator of inflammation and nutritional status. As classic cellular effectors, neutrophils are crucial for mediating inflammatory responses (11). Albumin is a vital indicator of nutritional status and chronic inflammation in the body, with lower levels typically associated with chronic diseases and malnutrition (12). NPAR combines neutrophils and albumin to more comprehensively reflect the inflammatory and nutritional status of an individual (13). Studies have shown that high NPAR is associated with the risk of death from a variety of chronic diseases such as chronic obstructive pulmonary disease and cancer (14, 15). However, minimal studies are available regarding the correlation between NPAR and CVD, especially in large populations. Therefore, this study investigates the association between NPAR and CVD based on large-scale population data from the NHANES database, aiming to validate the efficacy of NPAR as a potential CVD biomarker.

## 2 Materials and methods

### 2.1 Study design

This study used a retrospective cross-sectional study design to explore the association between NPAR and the prevalence of CVD in U.S. adults using data from the National Health and Nutrition Examination Survey (NHANES) database (NHANES 2015–2016). The study included adults >18 years old with complete neutrophil percentage and albumin testing data, as well as diagnostic CVD information. The research excluded individuals who lacked key variable dataas, well as patients with a history of tumors and those with inflammatory diseases such as rheumatoid arthritis and osteoarthritis. The effect of potential covariates such as age, sex, race, body mass index (BMI), education, alcohol consumption, smoking, hypertension, and diabetes were also considered.

### 2.2 Study objects and data sources

**Figure 1:**
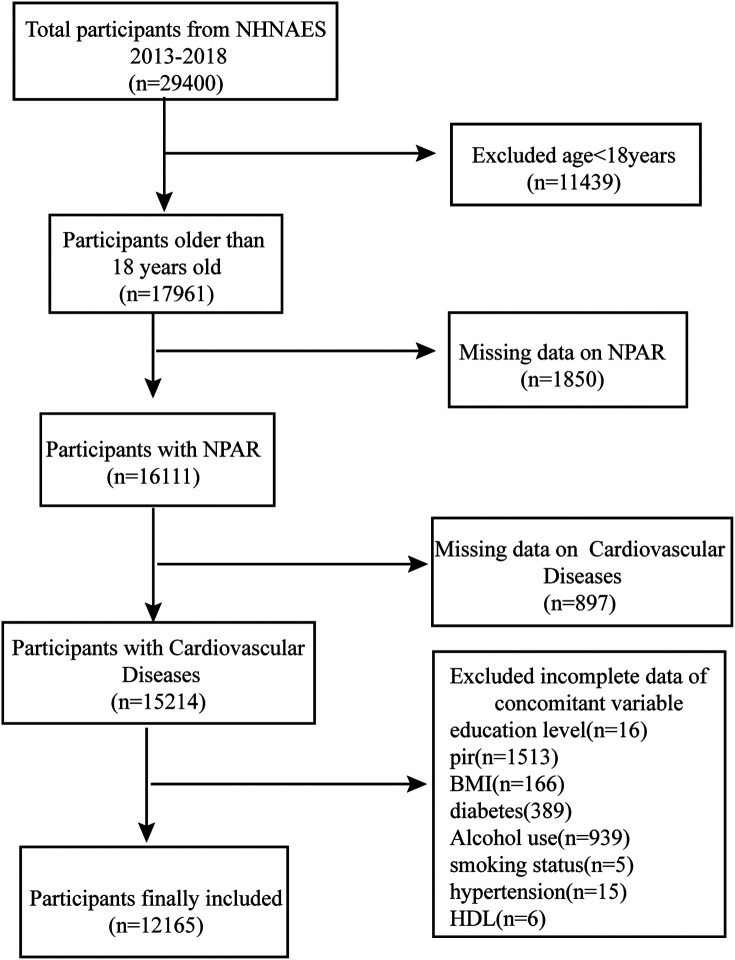
Flowchart of study participants selection.

The NHANES database employs multistage, random sampling for the annual collection of considerable data from the U.S. population, including information on demographic characteristics, lifestyle, health status, nutritional intake, physical examination, and laboratory tests. The data in the NHANES database are highly representative and reliable and are commonly used in disease epidemiology, nutrition, and environmental health research (16, 17). This study analyzed data from 2013 to 2018, including demographic characteristics (age, sex, race, education, and family poverty-to-income ratio (PIR), BMI, biochemical indicators (Total cholesterol, High-density lipoprotein), and other diagnostic information. All data were collected according to standardized NHANES processes and quality control procedures. A total of 29,400 participants were enrolled during 2013–2018. First, 11,439 individuals younger than 18 years old were excluded, after which the remaining 17,961 adult participants were screened to exclude those with missing NPAR (*n* = 1,850) and CVD data (*n* = 897). Also excluded were individuals with incomplete demographic information and missing covariate data (e.g., smoking status, BMI, and alcohol consumption, as well as hypertension and diabetes history). Ultimately, 12,165 participants were included in the study (Figure 1).

### 2.3 Assessment of the outcome variables

CVD status was determined according to self-reported physician diagnoses obtained during personal interviews using a standardized medical status questionnaire. Participants were asked, “Has a doctor or other health professional ever told you that you have congestive heart failure, coronary heart disease, angina, myocardial infarction, or a stroke?” Participants were considered as having CVD if they answered “yes” to any of these questions. Specific conditions such as congestive heart failure, myocardial infarction, angina pectoris, and coronary heart disease were also defined by answering “yes” to the corresponding questions above.

### 2.4 Exposure variables and covariates

This study used NPAR as an exposure variable, which represented the neutrophil percentage-to-albumin ratio (18, 19). To accurately determine the correlation between NPAR and CVD, several influencing covariates were examined, including age, sex, race, education level, BMI, PIR, smoking status, drinking behavior, diabetes, and hypertension. Sex was categorized as male/female, and race was categorized as Mexican American/Other Hispanic/Non-Hispanic White/Non-Hispanic Black/Non-Hispanic Asian/Other. The smoking status (yes/no) was categorized as smoking less than 100 cigarettes or smoking 100 or more cigarettes in a lifetime. The alcohol consumption frequency was defined as drinking less than 12 times per year or drinking 12 or more times per year. Diabetes and hypertension were categorized as yes/no, respectively. Information on each of these variables is available at www.cdc.gov/nchs/nhanegov/nchs/nhane.

### 2.5 Statistical analysis

First, the participants were categorized into CVD and non-CVD groups based on their diagnoses. The information of continuous variables was expressed as $\bar{X}\pm S$ and categorical variables were expressed as percentages. The chi-square (*χ*^2^) test is used for comparing two samples of categorical variables, while the independent samples *t*-test is used for comparing two samples of continuous variables. A multifactorial logistic regression model was used to investigate the relationship between NPAR and CVD. The logistic regression model was chosen because it is a widely used and well-established method for examining the association between a binary outcome (such as the presence or absence of CVD) and one or more predictor variables (in this case, NPAR). It provides odds ratios (OR) and confidence intervals (CI) that quantify the strength and significance of the association. Moreover, this method allows for the inclusion of multiple covariates, which helps to control for potential confounders that could influence the relationship between NPAR and CVD. Model 1 included adjustments for sex, age, and race, while Model 2 added adjustments for BMI, education level, PIR, alcohol and smoking habits, diabetes, hypertension, High-density lipoprotein (HDL) cholesterol, and total cholesterol. The nonlinear relationship between NPAR and CVD was explored via a restricted cubic spline (RCS) with 4 knots. Finally, subgroup analyses and interaction tests were performed to examine the potential differences between the genders in depth. Subsequently, subgroup analyses and interaction tests were performed to examine the potential differences among the covariates in depth (20). These analyses were essential for several reasons. First, subgroup analyses allow us to identify whether the association between NPAR and CVD varies across different demographic or clinical characteristics, such as gender. This approach helps to assess the robustness of our findings and identify potential subpopulations that may be at higher odds. Second, interaction tests were conducted to determine if specific factors, such as gender, modify the association between NPAR and CVD.All statistical analyses were performed using R language (version 4.2) and EmpowerStats software (version 6.0), while *p* < 0.05 was considered statistically significant.

## 3 Results

### 3.1 Baseline characteristics

Table 1 demonstrates the baseline demographic characteristics of the participants in this study. This included 12,165 individuals with a mean age of 49.54 ± 17.53 years, of whom 1,165 were diagnosed with CVD and 12,165 were not. Significant differences were evident between the CVD and non-CVD participants in terms of age, household PIR, neutrophils, albumin, BMI, HDL, total cholesterol, sex, race, education level, heart failure, coronary heart disease, angina pectoris, stroke, diabetes mellitus, smoking status, and hypertension (Table 1).

**Table 1:** Characteristics of participants.

Characteristic	Total	CVD	No-CVD	*P*-value
	12,165	1,165	11,000	
Age, year	49.54 ± 17.53	66.19 ± 12.35	47.77 ± 17.06	<0.001
PIR	2.52 ± 1.62	2.16 ± 1.46	2.56 ± 1.63	<0.001
Neutrophil percentage, %	57.48 ± 9.42	59.31 ± 10.09	57.28 ± 9.32	<0.001
Albumin, g/dl	4.21 ± 0.36	4.08 ± 0.35	4.22 ± 0.36	<0.001
BMI	29.54 ± 7.22	30.95 ± 7.71	29.39 ± 7.15	<0.001
HDL, mg/dl	53.44 ± 16.44	50.69 ± 16.25	53.73 ± 16.43	<0.001
Total cholesterol, mg/dl	189.30 ± 41.61	174.45 ± 42.59	190.87 ± 41.20	<0.001
Gender (male/female)				<0.001
Male	5,916 (48.63%)	653 (56.05%)	5,263 (47.85%)	
Female	6,249 (51.37%)	512 (43.95%)	5,737 (52.15%)	
Race				<0.001
Mexican American	1,753 (14.41%)	95 (8.15%)	1,658 (15.07%)	
Other hispanic	1,232 (10.13%)	105 (9.01%)	1,127 (10.25%)	
Non-hispanic white	4,876 (40.08%)	618 (53.05%)	4,258 (38.71%)	
Non-hispanic black	2,451 (20.15%)	253 (21.72%)	2,198 (19.98%)	
Other race	1,853 (15.23%)	94 (8.07%)	1,759 (15.99%)	
Education level				<0.001
Less than 9th grade	981 (8.06%)	116 (9.96%)	865 (7.86%)	
9–11th grade	1,410 (11.59%)	175 (15.02%)	1,235 (11.23%)	
High school graduate	2,801 (23.03%)	317 (27.21%)	2,484 (22.58%)	
Some college or AA degree	3,902 (32.08%)	371 (31.85%)	3,531 (32.10%)	
College graduate or above	3,071 (25.24%)	186 (15.97%)	2,885 (26.23%)	
Congestive heart failure (YES/NO)				<0.001
YES	399 (3.28%)	399 (34.25%)	0 (0.00%)	
NO	11,766 (96.72%)	766 (65.75%)	11,000 (100.00%)	
Coronary heart disease (YES/NO)				<0.001
YES	520 (4.27%)	520 (44.64%)	0 (0.00%)	
NO	11,645 (95.73%)	645 (55.36%)	11,000 (100.00%)	
Angina pectoris (YES/NO)				<0.001
YES	305 (2.51%)	305 (26.18%)	0 (0.00%)	
NO	11,860 (97.49%)	860 (73.82%)	11,000 (100.00%)	
Stroke (YES/NO)				<0.001
YES	453 (3.72%)	453 (38.88%)	0 (0.00%)	
NO	11,712 (96.28%)	712 (61.12%)	11,000 (100.00%)	
Diabetes (YES/NO)				<0.001
YES	1,798 (14.78%)	449 (38.54%)	1,349 (12.26%)	
NO	10,367 (85.22%)	716 (61.46%)	9,651 (87.74%)	
Alcohol use (YES/NO)				0.952
YES	9,396 (77.24%)	899 (77.17%)	8,497 (77.25%)	
NO	2,769 (22.76%)	266 (22.83%)	2,503 (22.75%)	
Smoking status (YES/NO)				<0.001
YES	5,265 (43.28%)	717 (61.55%)	4,548 (41.35%)	
NO	6,900 (56.72%)	448 (38.45%)	6,452 (58.65%)	
Hypertension (YES/NO)				<0.001
YES	4,451 (36.59%)	871 (74.76%)	3,580 (32.55%)	
NO	7,714 (63.41%)	294 (25.24%)	7,420 (67.45%)	

PIR, Poverty-to-income ratio; BMI, Body mass index; HDL, High-density lipoprotein.

### 3.2 Multifactorial logistic regression analysis of NPAR and CVD

Table 2 demonstrates the relationship between NPAR from the NHANES database and CVDs and their subtypes (heart failure, coronary heart disease, angina pectoris, and stroke) in different logistic regression models. The preliminary model (non-adjusted), which did not consider the effect of any confounding factors, showed a significant positive correlation between NPAR and the likelihood of developing these diseases. However, when potential confounders such as sex, age, and race were included for adjustment (Model 1), the association between NPAR and angina became insignificant. The correlation between NPAR and CVD, heart failure, and stroke remained significant, which was confirmed after further consideration of additional confounders (Model 2). Specifically, as a continuous variable, NPAR was associated with CVD at an OR of 1.03 (95% CI: 1.01–1.06, *P* = 0.0125), heart failure at an OR of 1.09 (95% CI: 1.05–1.13, *P* < 0.0001), and stroke at an OR of 1.06 (95% CI: 1.02–1.09, *P* = 0.0016). A comparison between the highest (Q4) and lowest (Q1) NPAR quartiles indicated increased odds of CVD (OR = 1.28, 95% CI:1.04–1.57, *P* = 0.0177), heart failure (OR = 1.66, 95% CI:1.18–2.34, *P* = 0.0035), and stroke (OR = 1.74, 95% CI:1.28–2.36, *P* = 0.0004).

**Table 2:** Association between NPAR and cardiovascular diseases in logistic regression models from the NHANES.

Exposure	Non-adjusted model		Model 1		Model 2	
	OR (95%CI)	*P* value	OR (95%CI)	*P* value	OR (95%CI)	*P* value
Cardiovascular disease						
NPAR (continuous)	**1.13** **(1.11, 1.16)**	**<0**.**0.001**	**1.09** (**1.06, 1.11)**	**<0**.**0001**	**1.03** (**1.01, 1.06)**	**0**.**0125**
NPAR (quartile)						
Q1	1		1		1	
Q2	1.24 (1.02, 1.51)	0.0325	1.21 (0.98, 1.49)	0.0717	1.14 (0.92, 1.42)	0.2322
Q3	**1.60** (**1.32, 1.93)**	**<0**.**0001**	**1.35** (**1.10, 1.65)**	**0**.**0038**	1.13 (0.92, 1.40)	0.2481
Q4	**2.56** (**2.14, 3.06)**	**<0**.**0001**	**1.81** (**1.49, 2.19)**	**<0**.**0001**	**1.28** (**1.04, 1.57)**	**0**.**0177**
Congestive heart failure						
NPAR (continuous)	**1.20** (**1.16, 1.23)**	**<0**.**0001**	**1.16** (**1.12, 1.20)**	**<0**.**0001**	**1.09** (**1.05, 1.13)**	**<0**.**0001**
NPAR (quartile)						
Q1	1		1		1	
Q2	1.32 (0.91, 1.90)	0.1409	1.30 (0.89, 1.89)	0.1731	1.22 (0.83, 1.79)	0.3198
Q3	**2.06** (**1.46, 2.89)**	**<0**.**0001**	**1.77** (**1.25, 2.51)**	**0**.**0013**	1.41 (0.99, 2.02)	0.0593
Q4	**3.64** (**2.65, 4.98)**	**<0**.**0001**	**2.60** (**1.87, 3.61)**	**<0**.**0001**	**1.66** (**1.18, 2.34)**	**0**.**0035**
Coronary heart disease						
NPAR (continuous)	1.14 (1.11, 1.17)	<0.0001	1.07 (1.04, 1.11)	<0.0001	1.03 (0.99, 1.06)	0.1543
NPAR (quartile)						
Q1	1		1		1	
Q2	1.28 (0.95, 1.71)	0.1050	1.16 (0.85, 1.57)	0.3497	1.10 (0.81, 1.51)	0.5350
Q3	1.51 (1.13, 2.00)	0.0046	1.13 (0.84, 1.52)	0.4248	0.96 (0.71, 1.31)	0.8076
Q4	2.70 (2.08, 3.50)	<0.0001	1.59 (1.21, 2.10)	0.0010	1.18 (0.88, 1.57)	0.2656
Angina pectoris						
NPAR (continuous)	1.10 (1.06, 1.14)	<0.0001	1.04 (1.00, 1.09)	0.0595	0.99 (0.95, 1.03)	0.5691
NPAR (quartile)						
Q1	1		1		1	
Q2	1.52 (1.06, 2.19)	0.0244	1.39 (0.96, 2.01)	0.0806	1.30 (0.89, 1.90)	0.1673
Q3	1.71 (1.20, 2.45)	0.0031	1.34 (0.93, 1.93)	0.1172	1.12 (0.77, 1.63)	0.5426
Q4	2.05 (1.45, 2.90)	<0.0001	1.31 (0.91, 1.87)	0.1413	0.91 (0.63, 1.32)	0.6358
Stroke						
NPAR (continuous)	**1.13** (**1.09, 1.16)**	**<0**.**0001**	**1.09** (**1.06, 1.13)**	**<0**.**0001**	**1.06** (**1.02, 1.09)**	**0**.**0016**
NPAR (quartile)						
Q1	1		1		1	
Q2	1.34 (0.97, 1.85)	0.0755	1.36 (0.98, 1.89)	0.0684	1.31 (0.94, 1.84)	0.1088
Q3	**1.88** (**1.39, 2.55)**	**<0**.**0001**	**1.70** (**1.24, 2.32)**	**0**.**0009**	**1.55** (**1.13, 2.13)**	**0**.**0070**
Q4	**2.78** (**2.09, 3.70)**	**<0**.**0001**	**2.11** (**1.56, 2.84)**	**<0**.**0001**	**1.74** (**1.28, 2.36)**	**0**.**0004**

Non-adjusted model:covariates were not adjusted; Model 1:Adjusted for gender, age, race; Model 2:Adjusted for gender, age, race, education level, PIR, BMI, diabetes, Alcohol use, smoking status, hypertension, HDL, total cholesterol. Bold values indicate statistical significance (*p* < 0.05).

### 3.3 Utilizing RCS analysis to explore the potential link between NPAR and various CVD outcomes

**Figure 2:**
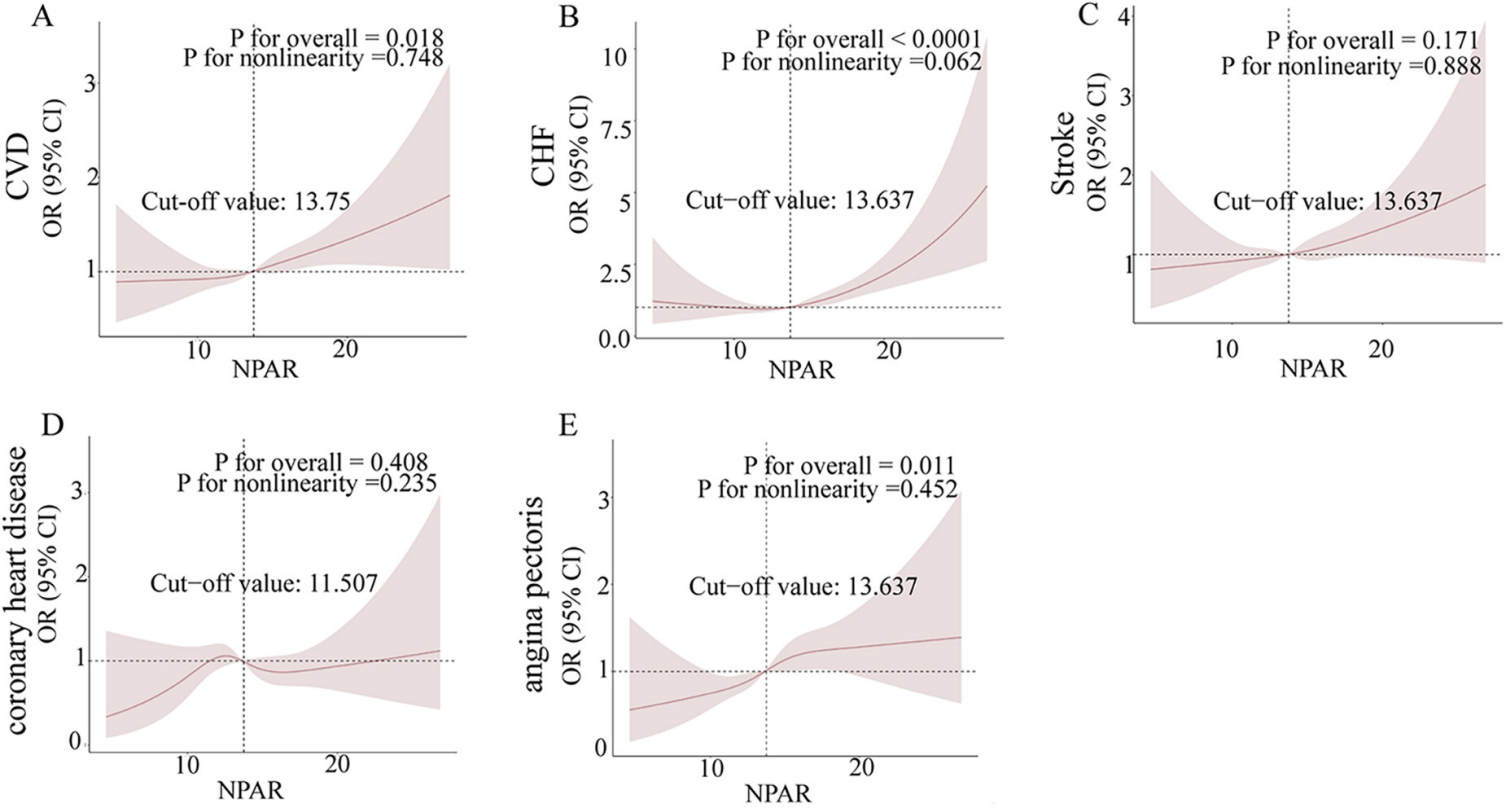
Non-parametric regression curve smoothing (RCS) analysis of the relationship between NPAR and cardiovascular diseases (CVD). Figures **(A)–(E)** present the RCS analysis results for the associations between NPAR and CVD, heart failure, stroke, coronary heart disease, and angina, respectively. A non-linear *p*-value of less than 0.05 suggests a significant non-linear relationship.

In this investigation, we employed restricted cubic splines (RCS) to rigorously analyze the relationships between the neutrophil-to-albumin ratio (NPAR) and various cardiovascular diseases, namely congestive heart failure (CHF), stroke, coronary heart disease (CHD), and angina pectoris. Figures 2A–E illustrate the correlation between NPAR and the associated with these conditions. Figure 2A highlights a significant positive correlation between NPAR and CVD odds (*P* for overall = 0.018), Figure 2B confirms a robust positive relationship with CHF (*P* for overall < 0.0001), and Figure 2E indicates a significant positive association with angina pectoris (*P* for overall = 0.011). Although nonlinearity in CHF is nearing significance in Figure 2B (*P* for nonlinearity = 0.062), the overall relationships between NPAR and these cardiovascular conditions tend to show a linear trend. Conversely, the correlations with stroke in Figure 2C (*P* for overall = 0.171) and CHD in Figure 2D (*P* for overall = 0.408) did not achieve statistical significance, and their nonlinear patterns were not marked.

### 3.4 Subgroup analysis

**Figure 3:**
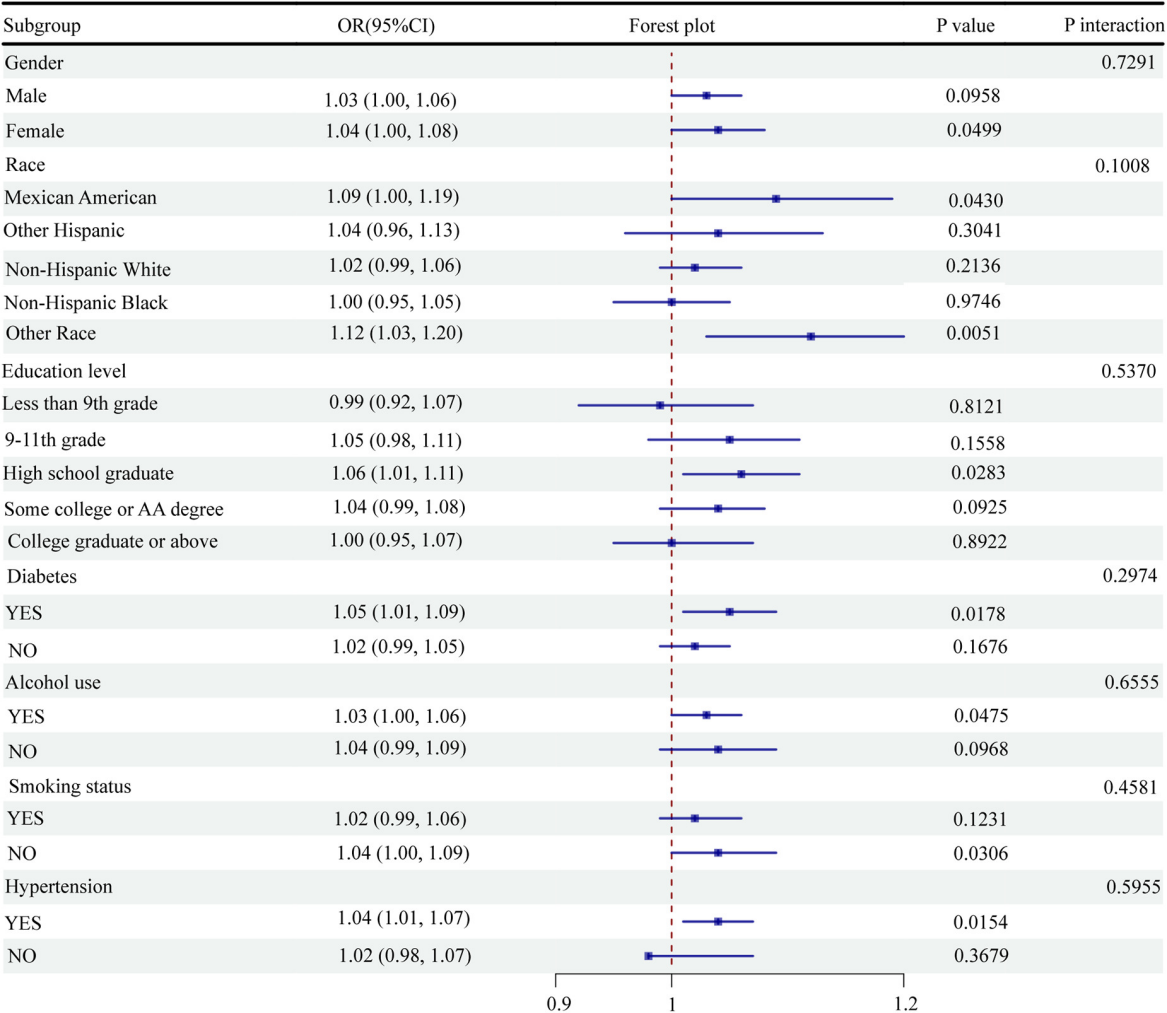
Subgroup analysis of the association between NPAR and CVD.

This study performed subgroup analyses and interaction tests between the NPAR levels and CVD odds. The results showed a statistically significant association between the NPAR levels and CVD odds in women (OR = 1.04, 95% CI: 1.00–1.08, *P* = 0.0499), Mexican Americans (OR = 1.09, 95% CI: 1.00–1.19, *P* = 0.0430), high school graduates (OR = 1.06, 95% CI: 1.01–1.11, *P* = 0.0283), diabetic patients (OR = 1.05, 95% CI: 1.01–1.09, *P* = 0.0178), alcohol consumers (OR = 1.03, 95% CI: 1.00–1.06, *P* = 0.0475), nonsmokers (OR = 1.04, 95% CI: 1.00–1.09, *P* = 0.0306), and hypertensive individuals (OR = 1.04, 95% CI: 1.01–1.07, *P* = 0.0154) (Figure 3). However, none of the interactions regarding sex, race, education level, diabetic status, alcohol consumption status, smoking status, and hypertension status reached statistical significance (Figure 3). Overall, the findings indicated a significant correlation between the NPAR levels and CVD odds in specific subgroups. However, the interaction between these factors was not significant, suggesting the broad applicability of the NPAR level as a predictor of CVD odds.

## 4 Discussion

This study examined the relationship between NPAR and CVD using data from the NHANES. The results showed that higher NPAR levels are significantly associated with an increased CVD odds, particularly for heart failure and stroke. Specifically, after adjusting for confounders such as age, sex, race, education level, BMI, smoking status, alcohol consumption, diabetes, and hypertension, the CVD odds was 3% higher for every one-unit increase in NPAR. In addition, individuals in the highest NPAR quartile displayed a significantly higher odds of heart failure and stroke than those in the lowest quartile.

Previous studies have confirmed the relationship between inflammation, nutritional status, and CVD. However, the current study is one of the few to use NPAR (a combined marker of inflammation and nutritional status) to predict CVD odds. By integrating these two aspects, it creates a single marker for a more comprehensive assessment of individual inflammation and nutritional status. Therefore, the NPAR application is expected to provide new biomarkers for early CVD screening and prevention while enhancing the precision of clinical diagnosis and management. The utilization of NPAR is also useful for identifying populations with high odds and developing more effective interventions to reduce CVD morbidity and mortality.

Several studies have investigated the relationship between individual inflammatory markers, such as CRP, white blood cell count, and CVD risk. For example, elevated CRP levels are associated with a higher risk of myocardial infarction and stroke (21). Similarly, other studies have found that higher leukocyte counts predict coronary heart disease and stroke (22, 23). However, the limitations of these markers, including their lack of variability and specificity, highlight the need to find more reliable indicators (8).

Inflammation and nutritional status are key factors in CVD pathogenesis. NPAR, by integrating these two aspects of inflammation and nutritional status, can more comprehensively reflect the health condition of the body, and thus offers several advantages over traditional single markers such as CRP and white blood cell count. First, it provides a more comprehensive evaluation of the likelihood of cardiovascular events by integrating two key aspects of cardiovascular pathophysiology. Second, NPAR shows consistent predictive value across populations and disease conditions, and studies have linked it to acute kidney injury, severe sepsis, and cirrhosis (24–26).

In the context of CVD, this study supports and extends the findings of previous research, demonstrating significant associations between NPAR and a variety of CVD outcomes (27). Specifically, the stronger association with heart failure and stroke highlights the potential of NPAR as a specific marker for these diseases, which may be due to the enhanced inflammatory response and nutritional depletion common in these patients (28, 29).

Furthermore, studies have shown that inflammation and malnutrition play a key role in CVD progression. For example, one study highlighted the central role of inflammation in atherosclerosis (30). A meta-analysis found that the risk of ischemic heart disease, myocardial infarction, any stroke, and ischemic stroke increased by 1.17-fold, 1.25-fold, 1.37-fold, and 1.46-fold, respectively, for every 10 g/L reduction in plasma albumin (31). The current study confirmed these observations, suggesting that by combining inflammation and nutritional status, NPAR may more effectively predict CVD than traditional single markers.

Further studies showed that other integrative markers similar to NPAR, such as the neutrophil-to-lymphocyte ratio (NLR), also supported the utility of combining multiple biomarkers for risk stratification. NLR was associated with increased cardiovascular risk and poor prognosis (32–34). Recent studies highlight the utility of other inflammatory indices, such as the neutrophil-to-lymphocyte ratio (NLR), monocyte-to-lymphocyte ratio (MLR), systemic immune-inflammation index (SII), and systemic inflammation response index (SIRI), in predicting cardiovascular outcomes (35–37). While NLR and SII focus on cellular immune responses, NPAR uniquely integrates inflammation and nutritional status. This distinctive feature further validates the use of NPAR as a composite marker and highlights its potential advantages over other indices in clinical practice. This further validates the use of NPAR as a composite marker and highlights the potential benefits of such measures in clinical practice. Another observational study demonstrated that higher neutrophil counts were significantly associated with higher cardiovascular risk using a Mendelian randomization study (MR) (38). Other research showed that elevated hereditary CRP levels were associated with ischemic vascular disease (39). These studies emphasized the role of inflammation in CVD, reinforcing the relevance of the current research findings regarding NPAR.

This study theoretically provides new evidence for utilizing NPAR as a CVD biomarker, enriches the etiological examination of CVD, and enhances the understanding of the role of inflammation and nutritional status in CVD development. In practice, these results suggest that NPAR could be used in likelihood assessment or screening guidelines. Specifically, it can provide new diagnostic and predictive clinical tools, assisting physicians in better assessing the likelihood of cardiovascular events in their patients and developing individualized treatment and prevention strategies. In addition, NPAR can also be combined with other known cardiovascular disease risk biomarkers to construct more accurate risk assessment models.

## 5 Research limitations

This study also presented several limitations. First, causality could not be determined since the data were derived from the NHANES cross-sectional study. Second, this study failed to incorporate all possible confounders. Several potential confounders remain that may have influenced the results. Third, the findings of this study are based on a U.S. population, and selection bias may not be directly generalizable to populations of other races or regions. Fourth, the calculation of NPAR relies on the accurate measurement of neutrophil percentage and serum albumin levels. Variations in laboratory techniques or systematic errors in measuring these parameters could introduce measurement bias. Finally, missing data for key variables, such as neutrophil percentage and albumin levels, may affect the study results. For example, if individuals with more severe health conditions are more likely to have missing data, this could lead to an underestimation of the true association between NPAR and CVD.

## 6 Conclusions

This study emphasizes the potential of NPAR as a predictive marker of CVD in a large, diverse population. Higher NPAR levels are significantly associated with higher CVD odds, particularly heart failure and stroke. Future research should focus on longitudinal studies to confirm these findings and explore the underlying mechanisms, aiming to incorporate NPAR into clinical practice to improve CVD odds assessment and management.

## Data Availability

The original contributions presented in the study are included in the article/Supplementary Material, further inquiries can be directed to the corresponding author.

## References

[1] World Health Organization. Cardiovascular diseases (CVDs) (2017). Available at: https://www.who.int/cardiovascular_diseases/en/ (Accessed September 4, 2024).

[2] Benjamin EJ Muntner P Alonso A Bittencourt MS Callaway CW Carson AP Heart disease and stroke statistics-2019 update: a report from the American Heart Association. Circulation. (2019) 139(10):e56–528. 10.1161/CIR.0000000000000659 30700139

[3] Virani SS Alonso A Benjamin EJ Bittencourt MS Callaway CW Carson AP Heart disease and stroke statistics-2020 update: a report from the American Heart Association. Circulation. (2020) 141(9):e139–596. 10.1161/CIR.0000000000000757 31992061

[4] Kondo T Nakano Y Adachi S Murohara T. Effects of tobacco smoking on cardiovascular disease. Circ J. (2019) 83(10):1980–5. 10.1253/circj.CJ-19-0323 31462607

[5] Strain WD Paldánius PM. Diabetes, cardiovascular disease and the microcirculation. Cardiovasc Diabetol. (2018) 17(1):57. 10.1186/s12933-018-0703-2 29669543 PMC5905152

[6] Yusuf S Hawken S Ôunpuu S Dans T Avezum A Lanas F Effect of potentially modifiable risk factors associated with myocardial infarction in 52 countries (the INTERHEART study): case-control study. Lancet. (2004) 364(9438):937–52. 10.1016/S0140-6736(04)17018-9 15364185

[7] Boyalla V Gallego-Colon E Spartalis M. Immunity and inflammation in cardiovascular disorders. BMC Cardiovasc Disord. (2023) 23(1):148. 10.1186/s12872-023-03185-z 36959565 PMC10035189

[8] Filipovic MG Luedi MM. Cardiovascular biomarkers: current Status and future directions. Cells. (2023) 12(22):2647. 10.3390/cells12222647 37998382 PMC10670787

[9] Ridker PM Hennekens CH Buring JE Rifai N. C-reactive protein and other markers of inflammation in the prediction of cardiovascular disease in women. N Engl J Med. (2000) 342(12):836–43. 10.1056/NEJM200003233421202 10733371

[10] Casas R Castro-Barquero S Estruch R Sacanella E. Nutrition and cardiovascular health. Int J Mol Sci. (2018) 19(12):3988. 10.3390/ijms19123988 30544955 PMC6320919

[11] Hansson GK. Inflammation, atherosclerosis, and coronary artery disease. N Engl J Med. (2005) 352(16):1685–95. 10.1056/NEJMra043430 15843671

[12] Arques S. Human serum albumin in cardiovascular diseases. Eur J Intern Med. (2018) 52:8–12. 10.1016/j.ejim.2018.04.014 29680174

[13] Sun T Shen H Guo Q Yang J Zhai G Zhang J Association between neutrophil percentage-to-albumin ratio and all-cause mortality in critically ill patients with coronary artery disease. Biomed Res Int. (2020) 2020:8137576. 10.1155/2020/8137576 32934964 PMC7479485

[14] Lan CC Su WL Yang MC Chen SY Wu YK. Predictive role of neutrophil-percentage-to-albumin, neutrophil-to-lymphocyte and eosinophil-to-lymphocyte ratios for mortality in patients with COPD: evidence from NHANES 2011–2018. Respirology. (2023) 28(12):1136–46. 10.1111/resp.14589 37655985

[15] Ko CA Fang KH Tsai MS Lee YC Lai CH Hsu CM Prognostic value of neutrophil percentage-to-albumin ratio in patients with oral cavity cancer. Cancers (Basel). (2022) 14(19):4892. 10.3390/cancers14194892 36230814 PMC9564168

[16] Taylor PN Albrecht D Scholz A Gutierrez-Buey G Lazarus JH Dayan CM Global epidemiology of hyperthyroidism and hypothyroidism. Nat Rev Endocrinol. (2018) 14(5):301–16. 10.1038/nrendo.2018.18 29569622

[17] Johnson CL Dohrmann SM Burt VL Mohadjer LK. National health and nutrition examination survey: sample design, 2011–2014. Vital Health Stat. (2014) 2(162):1–33. 25569458

[18] Zawiah M Khan AH Farha RA Usman A Al-Ashwal FY Akkaif MA. Assessing the predictive value of neutrophil percentage to albumin ratio for ICU admission in ischemic stroke patients. Front Neurol. (2024) 15:1322971. 10.3389/fneur.2024.1322971 38361641 PMC10868651

[19] Liu CF Chien LW. Predictive role of neutrophil-percentage-to-albumin ratio (NPAR) in nonalcoholic fatty liver disease and advanced liver fibrosis in nondiabetic US adults: evidence from NHANES 2017–2018. Nutrients. (2023) 15(8):1892. 10.3390/nu15081892 37111111 PMC10141547

[20] Liu B Wang J Li YY Li KP Zhang Q. The association between systemic immune-inflammation index and rheumatoid arthritis: evidence from NHANES 1999–2018. Arthritis Res Ther. (2023) 25(1):34. 10.1186/s13075-023-03018-6 36871051 PMC9985219

[21] Ridker PM Cushman M Stampfer MJ Tracy RP Hennekens CH. Inflammation, aspirin, and the risk of cardiovascular disease in apparently healthy men. N Engl J Med. (1997) 336(14):973–9. 10.1056/NEJM199704033361401 9077376

[22] Madjid M Awan I Willerson JT Casscells SW. Leukocyte count and coronary heart disease: implications for risk assessment. J Am Coll Cardiol. (2004) 44(10):1945–56. 10.1016/j.jacc.2004.07.056 15542275

[23] Li J Imano H Yamagishi K Tanaka M Cui R Muraki I Leukocyte count and risks of stroke and coronary heart disease: the circulatory risk in communities study (CIRCS). J Atheroscler Thromb. (2022) 29(4):527–35. 10.5551/jat.60889 33746157 PMC9090484

[24] Wang B Li D Cheng B Ying B Gong Y. The neutrophil percentage-to-albumin ratio is associated with all-cause mortality in critically ill patients with acute kidney injury. Biomed Res Int. (2020) 2020:5687672. 10.1155/2020/5687672 32219136 PMC7049452

[25] Gong Y Li D Cheng B Ying B Wang B. Increased neutrophil percentage-to-albumin ratio is associated with all-cause mortality in patients with severe sepsis or septic shock. Epidemiol Infect. (2020) 148:e87. 10.1017/S0950268820000771 32238212 PMC7189348

[26] Du X Wei X Ma L Liu X Guo H Liu Y Higher levels of neutrophil percentage-to-albumin ratio predict increased mortality risk in patients with liver cirrhosis: a retrospective cohort study. Eur J Gastroenterol Hepatol. (2023) 35(2):198–203. 10.1097/MEG.0000000000002470 36472501 PMC9770107

[27] Wang X Zhang Y Wang Y Liu J Xu X Liu J The neutrophil percentage-to-albumin ratio is associated with all-cause mortality in patients with chronic heart failure. BMC Cardiovasc Disord. (2023) 23(1):568. 10.1186/s12872-023-03472-9 37980510 PMC10657562

[28] Fichtlscherer S Heeschen C Zeiher AM. Inflammatory markers and coronary artery disease. Curr Opin Pharmacol. (2004) 4(2):124–31. 10.1016/j.coph.2004.01.002 15063355

[29] Uthamalingam S Patvardhan EA Subramanian S Ahmed W Martin W Daley M Utility of the neutrophil to lymphocyte ratio in predicting long-term outcomes in acute decompensated heart failure. Am J Cardiol. (2011) 107(3):433–8. 10.1016/j.amjcard.2010.09.039 21257011

[30] Gusev E Sarapultsev A. Atherosclerosis and inflammation: insights from the theory of general pathological processes. Int J Mol Sci. (2023) 24(9):7910. 10.3390/ijms24097910 37175617 PMC10178362

[31] Ronit A Kirkegaard-Klitbo DM Dohlmann TL Lundgren J Sabin CA Phillips AN Plasma albumin and incident cardiovascular disease: results from the CGPS and an updated meta-analysis. Arterioscler Thromb Vasc Biol. (2020) 40(2):473–82. 10.1161/ATVBAHA.119.313681 31852221

[32] Bagyura Z Kiss L Lux Á Csobay-Novák C Jermendy ÁL Polgár L Neutrophil-to-lymphocyte ratio is an independent risk factor for coronary artery disease in central obesity. Int J Mol Sci. (2023) 24(8):7397. 10.3390/ijms24087397 37108560 PMC10138538

[33] Tangjitgamol S Udayachalerm W Wanishsawad C Kaewwanna W Ativanichayapong N. Association of neutrophil-to-lymphocyte ratio and platelet-to-lymphocyte ratio and coronary artery disease among the physicians. J Inflamm Res. (2024) 17:59–66. 10.2147/JIR.S447750 38197034 PMC10775702

[34] Arbel Y Finkelstein A Halkin A Birati EY Revivo M Zuzut M Neutrophil/lymphocyte ratio is related to the severity of coronary artery disease and clinical outcome in patients undergoing angiography. Atherosclerosis. (2012) 225(2):456–60. 10.1016/j.atherosclerosis.2012.09.009 23040448

[35] Liuizė Abramavičiūtė A Mongirdienė A Laukaitienė J. Relationship between inflammatory readings and the degree of coronary atherosclerosis (pilot study). J Clin Med. (2024) 14(1):122. 10.3390/jcm14010122 39797206 PMC11722419

[36] Bani Hani DA Alshraideh JA Saleh A Alduraidi H Alwahadneh AA Al-Zaiti SS. Lymphocyte-based inflammatory markers: novel predictors of significant coronary artery disease^✰,✰✰^. Heart Lung. (2025) 70:23–9. 10.1016/j.hrtlng.2024.11.006 39549307

[37] Urbanowicz T Michalak M Komosa A Olasińska-Wiśniewska A Filipiak KJ Tykarski A Predictive value of systemic inflammatory response index (SIRI) for complex coronary artery disease occurrence in patients presenting with angina equivalent symptoms. Cardiol J. (2024) 31(4):583–95. 10.5603/CJ.a2023.0033 37314004 PMC11374332

[38] Luo J Thomassen JQ Nordestgaard BG Tybjærg-Hansen A Frikke-Schmidt R. Neutrophil counts and cardiovascular disease. Eur Heart J. (2023) 44(47):4953–64. 10.1093/eurheartj/ehad649 37950632 PMC10719495

[39] Kuppa A Tripathi H Al-Darraji A Tarhuni WM Abdel-Latif A. C-Reactive protein levels and risk of cardiovascular diseases: a two-sample bidirectional Mendelian randomization study. Int J Mol Sci. (2023) 24(11):9129. 10.3390/ijms24119129 37298077 PMC10252732

